# Supplementary material for: Identification of Freezing-Responsive microRNAs and Their Targets in Chinese Jujube by Small RNA and Degradome Sequencing
Source: Int J Mol Sci. 2026 Sep 4;27(17):7912. doi: 10.3390/ijms27177912 (PMC13566890; doi:10.3390/ijms27177912)
Supplement: Supplementary file 1 [file ijms-27-07912-s001.zip › Supplementary Material S2/novel_miR_152.pdf]

Provisional ID	:	novel_miR_152
Score total	:	251.7
Score for star read(s)	:	3.9
Score for read counts	:	243.3
Score for mfe	:	2.9
Score for randfold	:	1.6
Score for cons. seed	:	
Total read count	:	489
Mature read count	:	403
Loop read count	:	0
Star read count	:	86

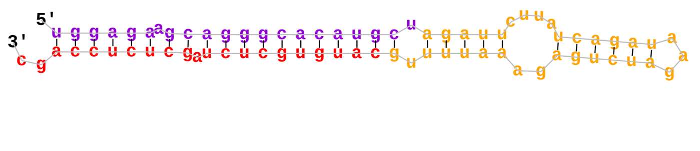

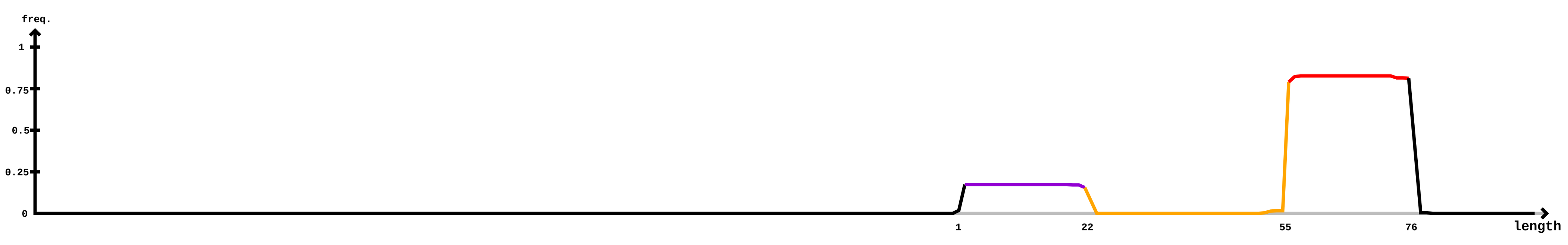


```
                                                                                                                                                                 Star                                                    Mature
5'- uucccauuauuaacuuuguugcugaaaaaaaaaaaacaaaaaugaaaaauugaccaugcuuucuguagaguuacuuguaggugaggugcccaauugaugauuuugaucauucagucagcagcaaaggauaauaggagaaggugaucagccaugcuggagaagcagggcacaugcuagauucuuaucagauaagaucugagaaauuuugcaugugcucuagcucuccagcuuggaucuucaucccuuacaag  -3'   obs
    uucccauuauuaacuuuguugcugaaaaaaaaaaaacaaaaaugaaaaauugaccaugcuuucuguagaguuacuuguaggugaggugcccaauugaugauuuugaucauucagucagcagcaaaggauaauaggagaaggugaucagccaugcuggagaagcagggcacaugcuagauucuuaucagauaagaucugagaaauuuugcaugugcucuagcucuccagcuuggaucuucaucccuuacaag        exp
    ((((.((((((..((((((((((((...........(((((.(..((((((((.(((((...(((....)))....))))).)))...)))))..).)))))..........)))))))))))))))))).))))..(((((....(((.((((((((.((((((((((((((.(((((....((((((...))))))..))))).))))))))))).)))))))))).)))....)))))......... reads  mm  sample
    .........................................................................................................................................................cuggagaagcagggcacGu...............................................................................  1   1   LT-1
    .........................................................................................................................................................cuggagaagcagggcacGugc.............................................................................  3   1   JCK-2
    .........................................................................................................................................................cuggagaagcagggcacGugc.............................................................................  4   1   JCK-3
    .........................................................................................................................................................cuggagaagcagggcacGugc.............................................................................  1   1   LT-2
    ..........................................................................................................................................................uggagaagcagggcacGugcu............................................................................  13  1   LT-1
    ..........................................................................................................................................................uggagaagcagggcacGugcu............................................................................  6   1   LT-3
    ..........................................................................................................................................................uggagaagcagggcacGugcu............................................................................  16  1   JT-1
    ..........................................................................................................................................................uggagaagcagggcacGugcu............................................................................  19  1   LCK-3
    ..........................................................................................................................................................uggagaagcagggcacaugcu............................................................................  1   0   LT-1
    ..........................................................................................................................................................uggagaagcagggcacGugcu............................................................................  10  1   LT-2
    ..........................................................................................................................................................uggagaagcagggcacaugcA............................................................................  1   1   JT-3
    ..........................................................................................................................................................uggagaagcagggcacGugcu............................................................................  1   1   JCK-1
    ..........................................................................................................................................................uggagaagcagggcacGugcu............................................................................  7   1   JCK-2
    ..........................................................................................................................................................uggagaagcagggcacaugcC............................................................................  1   1   JCK-2
    ..........................................................................................................................................................uggagaagcagggcacaugcA............................................................................  2   1   JT-2
    ............................................................................................................................................................................................................uuugcaugugcucuagcucuccagc......................  1   0   JCK-3
    ............................................................................................................................................................................................................uuugcaugugcucuagcucuccagc......................  1   0   JCK-1
    .............................................................................................................................................................................................................uugcaugugcucuagcucuccagc......................  3   0   JCK-2
    .............................................................................................................................................................................................................uugcaugugcucuagcucuccagc......................  2   0   JCK-3
    ..............................................................................................................................................................................................................ugcaugugcucuagcucuccagc......................  1   0   JCK-3
    ................................................................................................................................................................................................................caugugcucuagcucucc.........................  1   0   JCK-2
    ................................................................................................................................................................................................................caugugcucuagcucucc.........................  2   0   JCK-3
    ................................................................................................................................................................................................................caugugcucuagcucucc.........................  2   0   LCK-3
    ................................................................................................................................................................................................................caugugcucuagcucucc.........................  1   0   JCK-1
    ................................................................................................................................................................................................................caugugcucuagcucuccag.......................  1   0   LT-2
    ................................................................................................................................................................................................................caugugcuAuagcucuccagc......................  1   1   JCK-3
    ................................................................................................................................................................................................................caugugcucuagcucuccagc......................  93  0   JCK-3
    ................................................................................................................................................................................................................caugugcucuagcucuccagc......................  6   0   LT-2
    ................................................................................................................................................................................................................caugugcucuagcucuccagc......................  26  0   LT-3
    ................................................................................................................................................................................................................caugugcucuagcucuccagc......................  3   0   LT-1
    ................................................................................................................................................................................................................caugugcucuagcucuccagc......................  137 0   JCK-2
    ................................................................................................................................................................................................................caugugcucuagcucuccagc......................  96  0   JCK-1
    ................................................................................................................................................................................................................caugugcucuagcucuccagc......................  9   0   LCK-3
```

```
                                                                                                                                                                          Star                                                  Mature
uucccauuauuaacuuuguugcugaaaaaaaaaaaacaaaaaugaaaaauugaccaugcuuucuguagaguuacuuguaggugaggugcccaauugaugauuuugaucauucagucagcagcaaaggauaauaggagaaggugaucagccaugcuggagaagcagggcacaugcuagauucuuaucagauaagaucugagaaauuuugcaugugcucuagcucuccagcuuggaucuucaucccuuacaag
................................................................................................................................................................................................................caugugcucuGgcucuccagc......................  1  1  JCK-1
................................................................................................................................................................................................................caugugcuAuagcucuccagc......................  1  1  JCK-2
................................................................................................................................................................................................................caugugcucuagcucuccagcu.....................  2  0  JCK-1
................................................................................................................................................................................................................caugugcucuagcucuccagcu.....................  2  0  JCK-2
.................................................................................................................................................................................................................augugcucuagcucuccagc......................  2  0  JCK-3
.................................................................................................................................................................................................................augugcucuagcucuccagc......................  1  0  JCK-1
.................................................................................................................................................................................................................augugcucuagcucuccagc......................  4  0  LT-1
.................................................................................................................................................................................................................augugcucuagcucuccagc......................  1  0  LT-3
.................................................................................................................................................................................................................augugcucuagcucuccagc......................  8  0  JCK-2
..................................................................................................................................................................................................................ugugcucuagcucuccagcuug...................  1  0  LT-3
..................................................................................................................................................................................................................ugugcucuagcucuccagcuug...................  1  0  LT-1
```
